# Panoramic Stereo Imaging of a Bionic Compound-Eye Based on Binocular Vision

**DOI:** 10.3390/s21061944

**Published:** 2021-03-10

**Authors:** Xinhua Wang, Dayu Li, Guang Zhang

**Affiliations:** 1 School of Computer Science, Northeast Electric Power University, Jilin 132012, China; 2 State Key Laboratory of Applied Optics, Changchun Institute of Optics, Fine Mechanics and Physics, Chinese Academy of Sciences, Changchun 130033, China; lidayu@ciomp.ac.cn (D.L.); zhangguang0920@163.com (G.Z.)

**Keywords:** binocular stereo vision, panoramic imaging, stereo matching, depth information estimation

## Abstract

With the rapid development of the virtual reality industry, one of the bottlenecks is the scarcity of video resources. How to capture high-definition panoramic video with depth information and real-time stereo display has become a key technical problem to be solved. In this paper, the optical optimization design scheme of panoramic imaging based on binocular stereo vision is proposed. Combined with the real-time processing algorithm of multi detector mosaic panoramic stereo imaging image, a panoramic stereo real-time imaging system is developed. Firstly, the optical optimization design scheme of panoramic imaging based on binocular stereo vision is proposed, and the space coordinate calibration platform of ultra-high precision panoramic camera based on theodolite angle compensation function is constructed. The projection matrix of adjacent cameras is obtained by solving the imaging principle of binocular stereo vision. Then, a real-time registration algorithm of multi-detector mosaic image and Lucas-Kanade optical flow method based on image segmentation are proposed to realize stereo matching and depth information estimation of panoramic imaging, and the estimation results are analyzed effectively. Experimental results show that the stereo matching time of panoramic imaging is 30 ms, the registration accuracy is 0.1 pixel, the edge information of depth map is clearer, and it can meet the imaging requirements of different lighting conditions.

## 1. Introduction

In recent years, panoramic cameras are becoming more and more popular. Many well-known technology companies have released a variety of panoramic cameras, which have been successfully applied in the fields of street view mapping, virtual reality and video surveillance. Although there are many kinds of panoramic cameras at present, almost all of them provide 360° panoramic images without depth information. These panoramic images are composed of multiple two-dimensional images, which look flat and have no stereo sense of the real world [1,2]. In order to express clearly, these kind of two-dimensional panorama images are called monocular panorama. In this case, as like as two peas, the human eye sees the same panoramic image. So how to capture high-definition panoramic video with depth information and real-time stereo display has become a key technical problem to be solved. 

Panoramic stereo imaging system can provide close to real stereo visual effect without complex three-dimensional modeling, which is more convenient and efficient. The existing panoramic stereo imaging technology includes curved mirror [3], fish eye lens [4] and bionic compound-eye [5]. The curved mirror and fisheye lens have the defects of large distortion and low resolution, which limit their application. Biomimetic compound-eye is divided into two categories: micro-lens array and camera array. It has the advantages of compact structure, large field of view, high resolution, small distortion and high dynamic sensitivity. However, the focus of the work is mainly on image acquisition [6,7] and image mosaic [8], lacking consideration and utilization of 3D scene information. How to capture high-definition panoramic stereoscopic video that can record depth information, and how to process these stereoscopic video images in real time have become urgent problems to be solved. In order to meet the needs of engineering application, this paper selects the bionic compound-eye panoramic imaging technology based on a camera array as the research object, and proposes a bionic compound-eye panoramic real-time imaging system based on binocular vision, combined with the technologies of binocular stereo vision three-dimensional reconstruction, multi camera image synchronous acquisition and synchronous exposure control, and stereo video image real-time splicing and fusion, It can effectively solve the problems of stereo sense of panoramic imaging, poor real-time image processing and low stereo matching accuracy. The main contributions of this paper are as follows:

(1) Based on binocular stereo vision optimization, a binocular stereo vision optimization scheme is proposed. Firstly, the design of the imaging system requires that all the light collected should be tangent to a circle with the pupil distance as the diameter, and the number of cameras, the radius of the disc, and the field of view angle of the lens should also meet the constraints of the equivalent pupil distance. Then, the imaging system takes two shots, the first one is the left eye panorama, the second one is the right eye panorama, and finally the binocular panoramic stereo image is synthesized.
(2) A real-time registration method of multi detector image mosaic based on hardware and software is proposed. Firstly, an ultra-high precision calibration platform based on theodolite angle compensation is developed to calibrate the spatial coordinates of camera detector at sub-pixel level and calculate the imaging overlap area of adjacent cameras. Then, an image registration algorithm based on GPU acceleration is proposed to complete the real-time stitching of image overlapping areas.

The rest of this paper is organized as follows: Some related works in panoramic stereo imaging of bionic compound-eye are reviewed in Section 2. The principles of binocular stereo vision imaging and the composition of panoramic stereo imaging system are introduced in Section 3. Then, we describe the proposed approach elaborately in Section 4. Related experiment results are presented and discussed in detail in Section 5. Finally, we present our conclusions in Section 6.

## 2. Related Work

In the development of biomimetic compound-eye imaging system based on camera arraya, a number of large field of view and high resolution biomimetic compound-eye imaging systems have been developed. In terms of the development of scientific detection imaging equipment, a large field of view ultra-high pixel imaging system (aware-2) has been developed by the Duke University in the United States [9]. The system is composed of a concentric spherical lens and 98 micro camera arrays. The maximum output pixel is 1 billion. The horizontal field of view is 120° and the vertical field of view is 50° and the focal length is 35 mm. The system can clearly distinguish swans flying 1 km away. The research work is reported in detail in the journal *Nature* [10]. In addition, the chang’e-4 panoramic camera developed by Xi’an Institute of Optics and Precision Machinery of the Chinese Academy of Sciences, achieved stereo imaging of the lunar surface through the principle of binocular stereo vision [11]. Depending on the left and right rotation and up and down pitching of the mast, the panoramic imaging with large field of view and large range up and down can be realized. Then the panoramic three-dimensional image of the lunar surface can be obtained through image mosaic and three-dimensional inversion, so as to realize the scientific goals of three-dimensional optical imaging of the lunar surface, topography research, investigation and research of impact craters, analysis and comprehensive research of the lunar geological structure.

In the research and development of professional consumer products, panoramic cameras are mostly composed of several ordinary cameras arranged according to certain rules, and most of them are spherical or cylindrical in shape. The field of view of spherical panoramic cameras is 360° in all directions, such as the Nokia ozo, insta360, bubicam and sphericam. Cylindrical panoramic cameras only have 360° field of view in the horizontal direction, such as GoPro Odyssey, live planet, Facebook surround 360, jaunt one and Upano Xone. The Samsung Company of South Korea has launched a panoramic camera project where the image data collected in each frame is 350,000 pixels, and the camera looks like a flying saucer. It is equipped with 16 high-definition cameras, and is equipped with powerful processor and memory array. Each interval of two cameras constitutes a binocular stereo vision, which is responsible for the 45° field of view in the circumferential direction, and then realizes the 360° panoramic stereo imaging in the circumferential direction [12]. The current panoramic stereo imaging system still has many shortcomings, such as panoramic imaging visual effect flattening, immersion experience feeling is not strong, image processing real-time, unable to show real-time dynamic changes, image stereo matching accuracy is not high, the observer is prone to dizziness and so on [13,14].

## 3. Optical Design of Panoramic Stereo Imaging

### 3.1. Binocular Stereo Vision Model 

**Figure 1:**
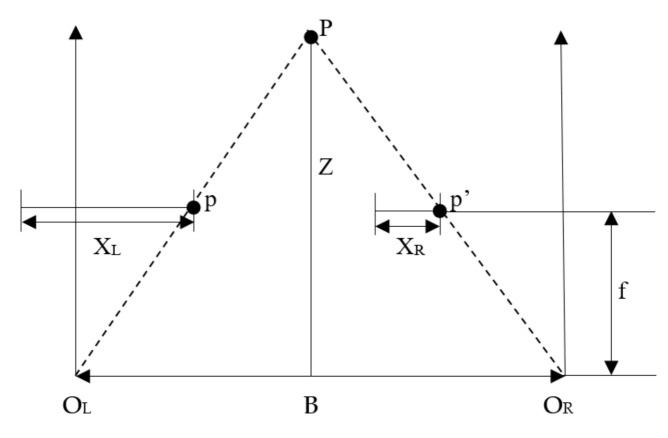
The stereo geometric model of binocular vision.

Binocular stereo vision is a method based on the principle of human parallax to obtain the three-dimensional information of the object. Two cameras are used to shoot the object from different angles, and the three-dimensional information of the object is recovered based on the principle of parallax. The main content of binocular parallax principle is through two viewpoints to observe the same object, to obtain the image of the same object in different perspectives, through the principle of triangulation to calculate the parallax between image pixels to obtain the three-dimensional spatial information of the object [15]. The geometric model of binocular stereo vision is shown in Figure 1. The geometric model includes two cameras with the same parameters. The optical axes of the two cameras are parallel to each other, and the X axes of the two cameras coincide with each other. In Figure 1, the geometric point P is the target object, and O_L_ and O_R_ represent the optical centers of the left and right cameras. The projection point of P on the projection plane of the left camera is p, and the projection point on the right camera is p’, and the horizontal coordinates of p and p’ in the pixel are X_L_ and X_R_, respectively. The distance between the camera and point P is Z, and the focal length of the camera is f. The distance d between the optical centers of two cameras is defined as the baseline distance.

According to the measurement principle of triangle similarity, the following relation can be obtained:

(1) $$\frac{Z-f}{Z}=\frac{B-\left(X_{L}-X_{R}\right)}{B}$$

(2) $$Z=\frac{B}{X_{L}-X_{R}}f=\frac{B}{d}f$$

where, *d* = *X_L_* − *X_R_*, is the difference of point *P* in the horizontal direction between the imaging point on the left and the imaging point on the right, that is, parallax. Therefore, each scene point in 3D space can recover its depth information by calculating its parallax. When the parallax values of all matching points in the left and right images are calculated, the parallax map is formed. The parallax map consists of a sequence of integer values. Each element in the sequence records the parallax values of the matching points. Matching disparity map is not only a visual method of disparity, but also a convenient method to observe the matching quality.

**Figure 2:**
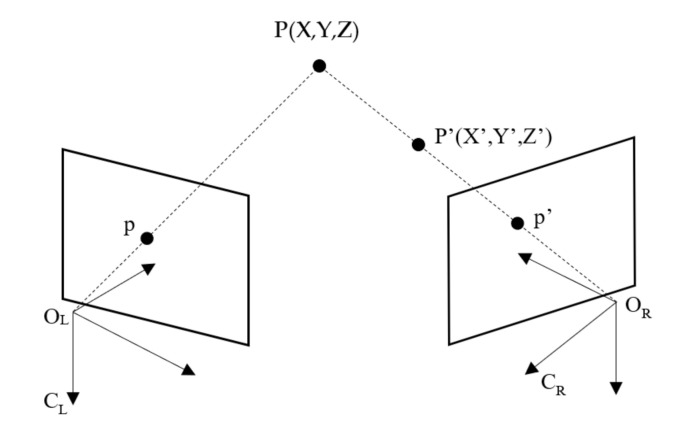
The basic principle of binocular stereo vision.

It can be seen from Equation (2) that the calculation of the depth information of the target object is not complicated, and it can be obtained smoothly only by knowing the baseline distance, the focal length of the camera and the parallax. The most difficult problem in stereo vision is the solution of the parallax map. The solution of disparity map needs to find out the corresponding points of the same point on the left and right images [16]. However, it is not easy to find the corresponding points in practical application. Some parameters of the camera are not known, and the position of the camera is not necessarily parallel. The geometric model of parallel binocular stereo in Figure 1 is a kind of project situation. Generally, the binocular vision model with arbitrary camera orientation is used, as shown in Figure 2.

In Figure 2, the imaging points of point P (X, Y, Z) on two cameras C_L_ and C_R_ are p and p’. If only one camera O_L_ is used to observe point P (X, Y, Z), the depth information of the point cannot be obtained, because on the line of O_R_P, the mapping points of any point P’ (X’, Y’, Z’) in the three-dimensional space are all p’, so the depth information of the corresponding point cannot be determined. If two cameras are used to observe point P (X, Y, Z) at the same time, point P is located on the line between O_L_P and O_R_P, which is the intersection of two rays. Therefore, the position of point P is uniquely determined. In this model, in order to determine P (X, Y, Z), it becomes to find p and p’ corresponding to P.

### 3.2. Camera Array Design of Panoramic Stereo Imaging

Compared with monocular panoramic image, if you want to get more realistic 3D 360 panoramic image, you need to calculate the parallax to get the image depth information. The panoramic stereo imaging system based on binocular vision uses two cameras with the same parameters to simulate the left and right eyes of human beings, and take photos at the same position to obtain the parallax. This requires that the imaging field of the two cameras should have enough overlapping parts to calculate the parallax. Therefore, it is necessary to use more cameras to take stereo photos in every direction of 360° around, and all the cameras should be in good condition. The camera must be strictly globally synchronized. In addition, the second panorama must be seamlessly stitched by the right eye camera. In this case, when viewing with VR glasses, two eyes see two different panoramas with parallax, which have the same sense of hierarchy in the virtual world as in the real world.

**Figure 3:**
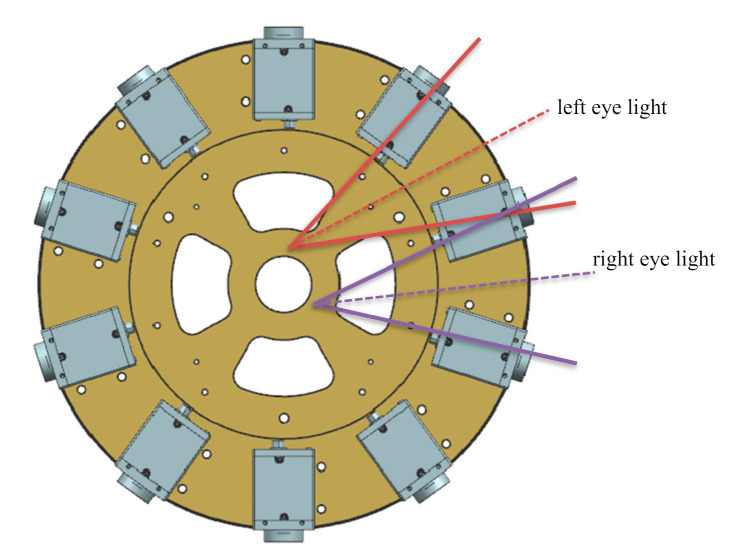
The panoramic stereo camera array based on binocular vision.

Camera array can be simply understood as light collection equipment, the intersection of the collected light and the imaging plane is the pixel. The general two-dimensional panorama requires all the light collected to converge at the central view, while the binocular stereo panorama requires two eyes to correspond to two different view positions. When the head rotates 360 degrees, the trajectory of two eyes is a circle with the diameter of pupil distance, and the binocular stereo panorama requires all the light collected to be tangent to this circle. The light collected by the left and right eyes intersect with the imaging plane to form the left eye panorama and the right eye panorama. The structure of panoramic stereo imaging camera array is shown in Figure 3.

Generally, all the light collected by the two-dimensional panoramic camera is required to converge at the central view, while the binocular stereo panoramic camera requires the left and right eyes to correspond to two different view positions, that is, the two points on the innermost circle in Figure 3. When the head rotates 360 degrees, the trajectory of the two eyes is a circle with the diameter of pupil distance (the innermost circle in Figure 3). The binocular stereo panoramic camera requires all the light collected to be tangent to each other In this way, the light collected by the left and right eyes respectively intersects with the target plane of the detector, and finally stitches to form the left eye panorama and the right eye panorama. In addition, in order to obtain a better panoramic 3D display effect, the following constraints should be satisfied between the number of cameras n, the horizontal field angle of view of a single camera *θ*, the radius of the supporting disc R and the IPD of the human eye:

(3) $$2R\times \sin\left(\frac{\theta }{2}-\frac{360}{N}\right)\approx \mathrm{IPD}$$

**Figure 4:**
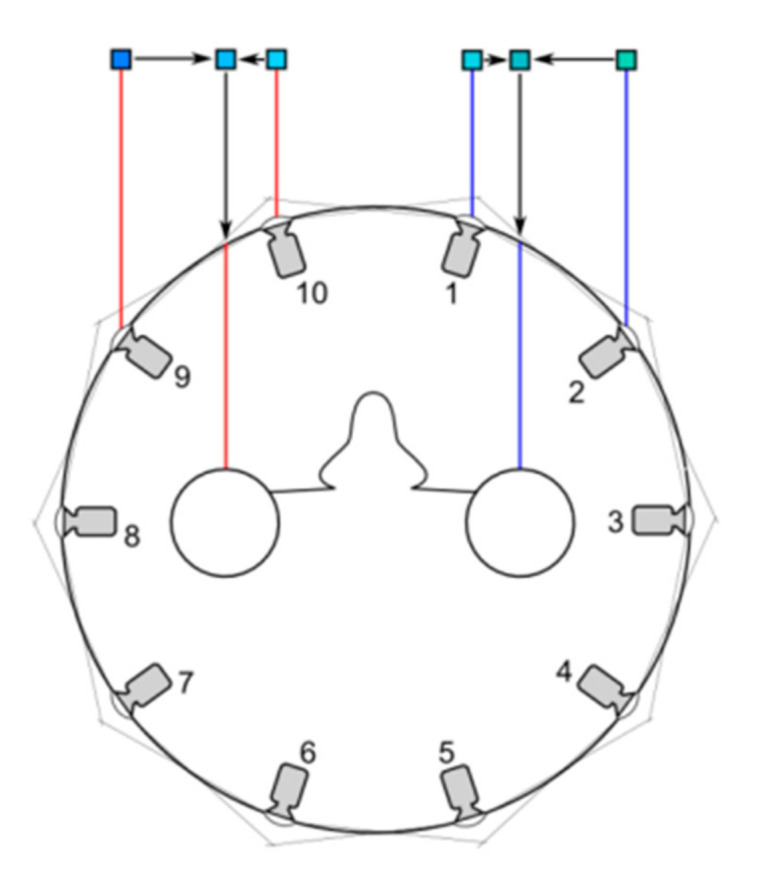
The stereoscopic vision of left and right eyes.

In order to produce normal stereopsis, the IPD should be at least 6.4 cm (the distance between ordinary eyes), so I > = 3.2 cm. Taking the panoramic camera developed in this paper as an example, n = 14, r = 15 cm, the horizontal field of view angle of the side camera is 90 ° and the horizontal field of view angle changes to 77° after barrel distortion correction. Equation (3) can get I = 3.32 cm > 3.2 cm, so it meets the 3D design requirements. This paper uses 10 cameras to illustrate how to synthesize a 3D visual image. As shown in Figure 4, the eye is in the circle, and the light thrown by the left eye is just between the two cameras. The pixels seen by the left eye are interpolated by the pixels of camera 9 and camera 10. The pixel value of camera 10 has a greater weight because the left eye ray is closer to camera 10 than camera 9. Similarly, the right eye pixel is calculated by interpolation of camera 1 and camera 2. Through this interpolation method, the number of cameras can be saved as much as possible.

**Figure 5:**
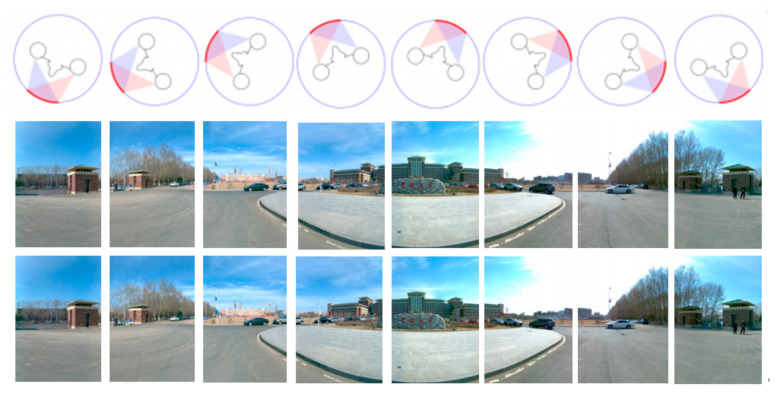
The calculation of stereoscopic images in different directions.

**Figure 6:**
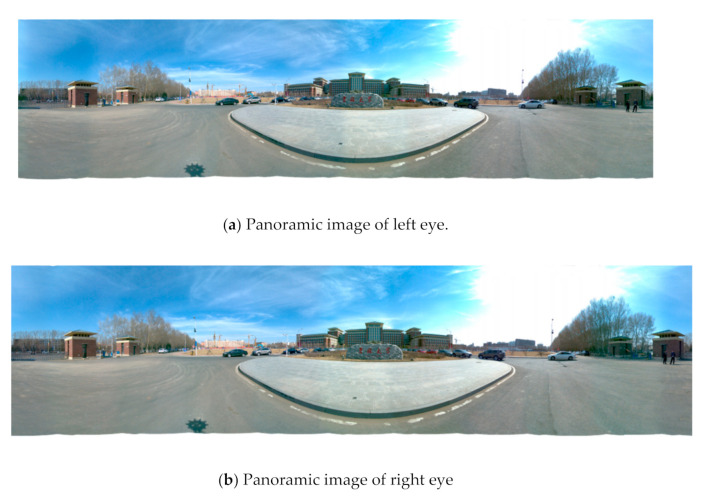
The left (**a**) and right eye (**b**) compose a stereoscopic panorama.

In Figure 4, if the emmetropia direction of the two eyes is changed, the views corresponding to the left and right eyes are also changed, as shown in Figure 5, that is, the stereoscopic images in different directions are synthesized. The stereo images in different directions are stitched to get the stereo panorama of left and right eyes, as shown in Figure 6.

## 4. Binocular Stereo Panoramic Image Synthesis Algorithm

**Figure 7:**
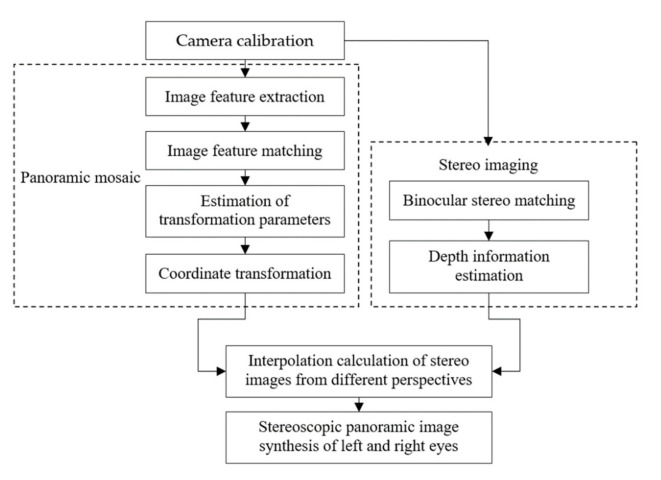
The binocular stereo panoramic image synthesis algorithm.

The implementation sequence of binocular stereo panoramic imaging synthesis algorithm is to obtain the binocular image first, then calibrate the camera, perform stereo matching, and finally perform depth information estimation. The binocular stereo panoramic image synthesis algorithm flow is shown in Figure 7. 

Camera calibration and stereo matching are the core parts of the algorithm. The purpose of camera calibration is to determine the basic relationship between the camera position and the target object, which will directly affect the accuracy of stereo matching and the efficiency of the algorithm. The purpose of stereo matching is to calculate the pixel matching relationship between the reference image and the target image, which will directly affect the result of depth information estimation. 

### 4.1. Ultra High Precision Camera Calibration Based on Binocular Stereo Vision 

Camera calibration is an important step of 3D scene reconstruction based on binocular stereo vision. The accuracy of camera calibration will directly affect the result of depth information estimation. Another function of calibration is to find the overlapping area of adjacent cameras, which can narrow the search range and improve the matching efficiency [17]. The calibration process of binocular stereo vision panoramic camera is as follows: firstly, based on the ultra-high precision calibration platform and binocular stereo vision camera calibration method, the ultra-high precision calibration of the spatial position coordinates of each camera optical axis in the panoramic imaging system is realized. Then, based on the calibration results of camera internal and external parameters, the rotation translation position relationship between adjacent cameras is solved, and the sub-pixel level calibration of binocular imaging overlapping area is realized.

**Figure 8:**
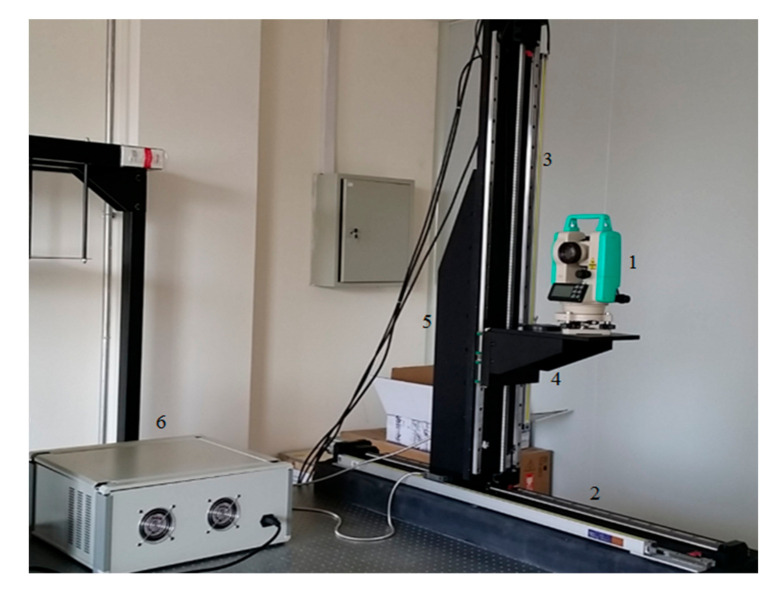
The ultra-high precision camera calibration platform.

In this paper, a high-precision calibration platform based on theodolite angle compensation function is constructed. The platform is mainly composed of two linear guides and a two-dimensional translation platform, a modified luminous theodolite and a servo controller. As shown in Figure 8, the parts are: 1-luminous theodolite (NEWLABS CO., LTD, Beijing, China), 2-horizontal guide rail (NEWLABS CO., LTD, Beijing, China), 3-vertical guide rail (NEWLABS CO., LTD, Beijing, China), 4-stage (NEWLABS CO., LTD, Beijing, China), 5-right angle fixed block (NEWLABS CO., LTD, Beijing, China), 6-servo controller (NEWLABS CO., LTD, Beijing, China). Firstly, the theodolite provides the infinite target source and emits the pattern with cross filament. Then, by solving the motion displacement equation, the two-dimensional translation table and theodolite are controlled to move to the specified position, and the cross wire is imaged at the center of each detector target surface respectively to complete the installation, adjustment and calibration of each detector. 

Considering the focal length of the imaging system and the pixel size of the detector, a 2′′ precision self-luminous theodolite is adopted, with the angle compensation range of ± 3′, and the focal length of the objective lens of 225 mm. The reticle is replaced by a cross wire with the width of 0.02 mm × 0.02 mm to ensure that the detector can find the cross wire, the fitting accuracy of centroid will not be affected by too large cross wires. In addition, because whether the center of the cross wire is aligned with the visual axis directly affects the accuracy of the calibration platform, it is necessary to adjust the alignment of the center of the cross wire with the visual axis through the forward and backward mirror method. Finally, in order to ensure the stability of the theodolite in the calibration process, three threaded holes are added to the base plate of the theodolite, which are aligned with the three through holes on the stage, and the theodolite is locked and fixed on the stage with bolts.

**Figure 9:**
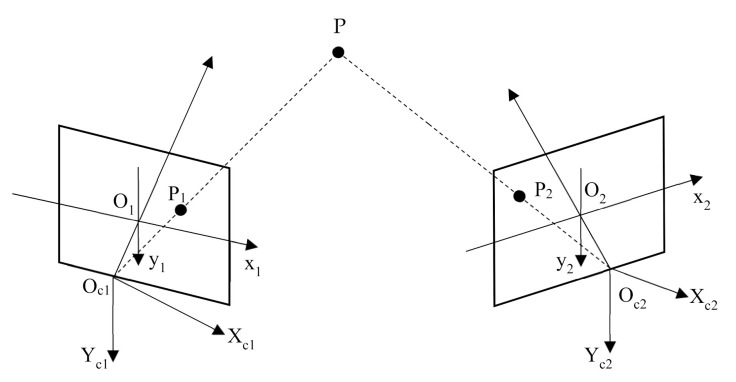
The coordinate calibration principle of binocular stereo vision.

For multi-camera stitching imaging system, in order to ensure that there is enough field of view overlap area between adjacent cameras for stitching and reduce the difficulty of image stitching and data processing in the later stage, it is necessary to calibrate the spatial position coordinates of camera detector accurately [18]. The calibration calculation process is as follows: Firstly, the theodolite provides the infinite target source and emits the pattern with cross filament. Then, by solving the motion displacement equation, the two-dimensional translation table and theodolite are controlled to move to the specified position, and the cross wire is imaged at the center of each detector target surface respectively to complete the installation, adjustment and calibration of each detector. In binocular stereo vision, it is necessary to calibrate the rotation and translation position relationship between two cameras, as shown in Figure 9.

Firstly, the internal and external parameters of the camera are calibrated by the ultra-high precision calibration platform, which are recorded as *R*_1_, *T*_1_ and *R*_2_, *T*_2_. *R*_1_ and *T*_1_ represent the position of the left camera relative to the world coordinate system, *R*_2_ and T_2_ represent the position of the right camera relative to the world coordinate system. For any point *P* existing in three-dimensional space, if its coordinates in the world coordinate system are *P_W_*, *P*_1_ in the left camera and *P*_2_ in the right camera, the following equation exists:

(4) $$\left\{\begin{array}{c} p_{1}=R_{1}P_{W}+T_{1}\\ p_{2}=R_{2}P_{W}+T_{2} \end{array}\right.$$

After eliminating *P_W_*, we can get:

(5) $$p_{2}=R_{2}R_{1}^{-1}p_{1}+T_{2}-R_{2}R_{1}^{-1}T_{1}=Rp_{1}+T$$

Let the relationship between the left and right cameras be rotation matrix *R* and translation matrix *T*, then:

(6) $$\left\{\begin{array}{c} R=R_{1}R_{1}^{-1}\\ T=T_{2}-R_{2}R_{1}^{-1}T_{1} \end{array}\right.$$

The rotation matrix *R* reflects the relative rotation angle of the two cameras, and the translation vector *T* reflects the distance between the two cameras.

### 4.2. Binocular Stereo Matching and Depth Information Estimation

In order to improve the stereo matching accuracy and meet the real-time requirements of image processing in engineering applications, a real-time binocular stereo matching algorithm based on hardware and software is proposed. The implementation steps of the algorithm are as follows:

*Step 1*: the 24-color standard calibration board is used to establish the color correction matrix to calibrate the image color consistency of multiple cameras. The distortion of the target image is calibrated by using the black-and-white chessboard. Read the preprocessed image data and expand each image by square projection.

*Step 2*: the imaging overlap region of the adjacent camera has been calibrated with the help of the ultra-high precision calibration platform, and the speed-up robust features (SURF) method has been used to extract the candidate feature points of the overlap region based on compute unified device architecture (CUDA) [19]. 

*Step 3*: the fast-approximate nearest neighbor (FANN) search algorithm has been accelerated by the CUDA basic linear algebra subroutines (CUBLAS) is proposed to obtain the initial matching points [20].

*Step 4*: the parallel progressive sample consensus (IPROSAC) algorithm based on interspersed interior point set is proposed to eliminate the false matching points [21].

**Figure 10:**
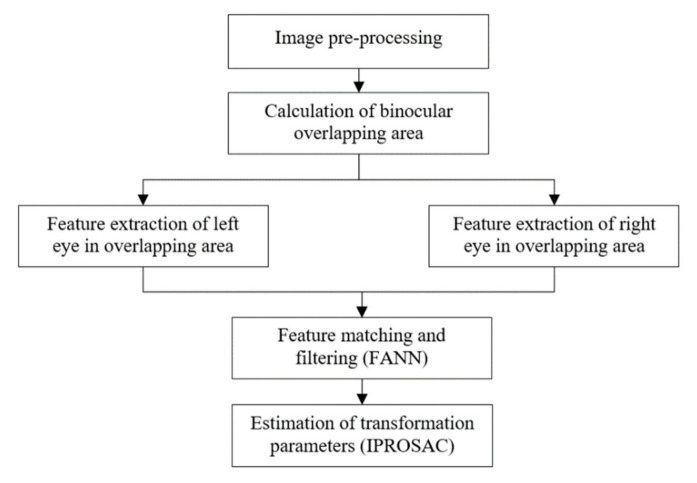
The stereo matching algorithm for binocular vision.

The algorithm flow is shown in Figure 10.

When the disparity map is obtained by stereo matching, the extraction of depth information is the key problem. In this paper, the Pyramid Lucas-Kanade optical flow method based on image segmentation was employed to extract depth information and several achievements were reached. Firstly, the number of required pyramid layers was determined through calculation of the maximum motion vector of the image, such adaptive determination was able to make up the losses of information caused by too many layers and to overcome the failure of Lucas-Kanade optical flow caused by too few layers. Secondly, the information acquired upon mean shift image segmentation in each layer was exploited to remove the error pixels of the motion vector at each iteration, therefore extraction of the depth information was more accurate. Thirdly, time complexity was reduced by adaptive adjustment of the number of iterations in each layer, while the quality of the experimental results remained nearly unchanged. Lastly, the depth map was optimized through segmenting by statistics the depths in each class to make the final edge information much clearer, which enhanced the rendered 3D effect.

**Figure 11:**
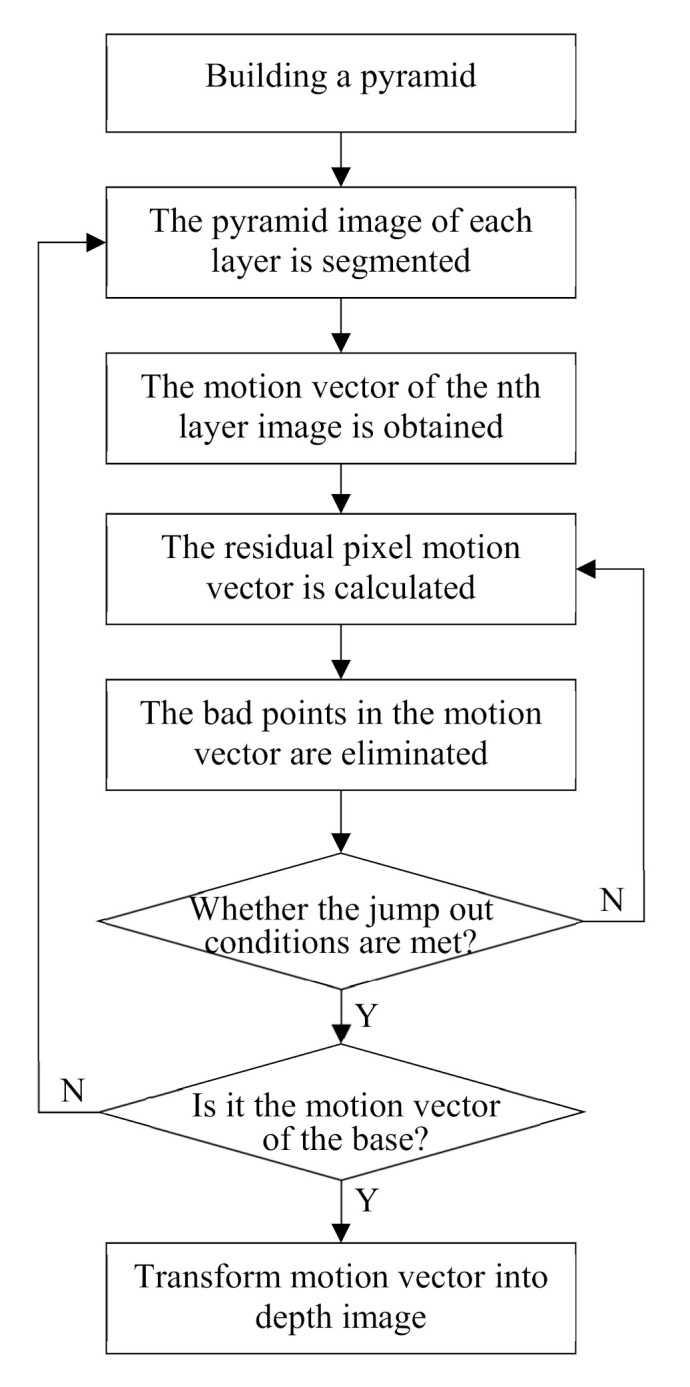
The pyramid Lucas-Kanade optical flow algorithm based on image segmentation.

The Lucas-Kanade optical flow method uses spatial brightness gradient information to obtain better matching position. It has three assumptions [22,23]: firstly, the brightness between two adjacent frames is constant; secondly, in order to solve the aperture problem, there is the same motion in the same integration window; thirdly, the motion of the object between adjacent frames is relatively small. In order to make all kinds of video images generally conform to the hypothesis thirdly, Bouguet proposed pyramid Lucas Kanade optical flow algorithm. This paper improves and optimizes the method, and proposes pyramid Lucas-Kanade optical flow algorithm based on image segmentation. Its basic idea is: using pyramid Lucas-Kanade optical flow algorithm to get the motion vector through gradient matching, and then segmenting each layer of pyramid image by Mean Shift. Using the segmented image information to optimize the motion vector, the bad image in the depth map is reduced In order to improve the quality of depth image, the number of points is reduced obviously. The algorithm flow is shown in Figure 11.

The Pyramid Lucas-Kanade optical flow algorithm based on image segmentation uses mean shift image segmentation algorithm to obtain image segmentation information. Its basic principle is: firstly, calculate the offset mean value of the current point, move the point to its offset mean value, and then take it as a new initial starting point, continue to move until certain conditions are met. After segmentation, even pixels with similar color and distance are classified into one category. This algorithm assumes that the same kind of pixels have the same motion vector, which is more in line with the actual situation than the same integration window has the same motion vector [24]. The main steps are as follows:

*Step 1*: according to the number of layers of the image, the parameters of mean shift image segmentation are determined to obtain the image segmentation information.

*Step 2*: calculate the mean value of motion vectors in the same class.

*Step 3*: make the difference between the motion vectors in this class and the mean value, and calculate the mean value of all motion vectors in this class whose absolute value is less than a certain threshold.

*Step 4*: the motion vector whose absolute value is greater than this threshold is regarded as a bad point. Change the value of the bad point to the mean value calculated in step 3 to enter the next iteration.

In addition, Lucas-Kanade optical flow method uses Newton iteration method to get better matching points. After the initial motion vectors are calculated in each layer of pyramid, the motion vectors of the residual pixels are calculated iteratively. The motion vectors of the residual pixels are added to the initial motion vectors, and the better results are obtained by iteratively updating [25]. In this algorithm, the number of adaptive iterations is proposed, so that the number of iterations is slightly different, and the time cost can be reduced when the quality of the depth image is almost the same. The main idea of the algorithm is that when the motion vectors of participating pixels calculated by each iteration are less than a certain threshold, the next iteration will not be carried out. In addition, this algorithm also provides an iteration jump out condition: Hessian matrix (A^T^A) is taken as an evaluation coefficient, and compared with the evaluation coefficient of the previous iteration, if the change is small, the next iteration will not be carried out, According to the judging condition of Equation (7):

*U*_r_ < 10^−2^ && *rcond*(A^T^A)_k_-*rcond*(A^T^A)_k−1_ < 10^−5^ (7)

where *U*_r_ is the motion vector of the residual pixels and k is the number of iterations. According to Equation (8), the final motion vector is transformed into depth image:

(8) $$depth=\frac{\sqrt{u^{2}+v^{2}}}{MV}\times 255$$

where, *MV* is the largest motion vector in the video frame, *u* and *v* are the horizontal and vertical motion vectors.

## 5. Results and Discussion

### 5.1. Testing Environment

**Figure 12:**
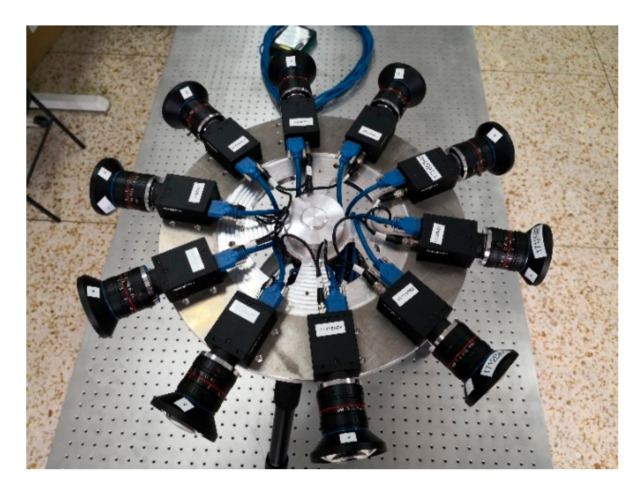
The binocular panoramic stereo imaging system.

In the aspect of image data acquisition, all the images are obtained by the binocular panoramic stereo imaging system, as show in Figure 12. the imaging system is composed of 10 cameras with the same specifications and models. the parameters of the imaging system are field of view 360°, number of pixels 30 million, and frame rate 30 FPS. Single camera parameters: field of view 73°, focal length 2.8 mm, sensor type CMOS CMV4000-3E5, resolution 2048 × 2048.

In the aspect of image data processing, the hardware and software environment of the computer are as follows: CPU Intel Core i9-9900k, GPU Geforce RTX 2080 Ti, RAM 64G, operating system Windows10 (64 bit), program development environment Matlab R2019b.

### 5.2. Experiments for Camera Calibration

**Figure 13:**
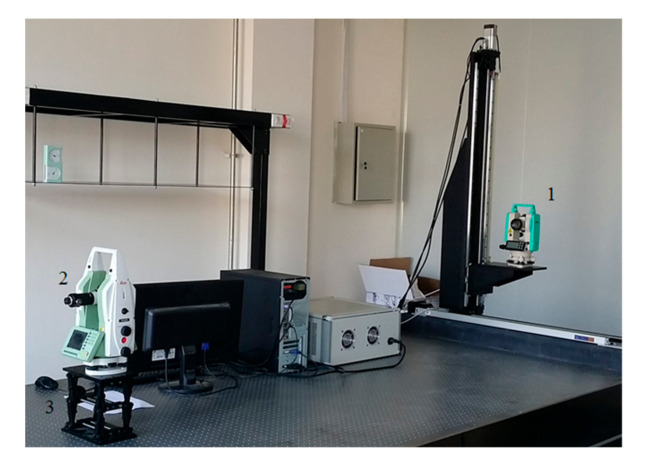
Alignment error testing experiment for four-axes calibration table.

In order to verify the alignment accuracy of the four-dimensional calibration platform, the self-calibration theodolite is used to carry out the alignment error detection experiment. The experiment is shown in Figure 13. In the figure, 1-four-dimensional calibration platform (NEWLABS CO., LTD, Beijing, China), 2-self calibration theodolite (NEWLABS CO., LTD, Beijing, China) and 3-lifting platform(NEWLABS CO., LTD, Beijing, China).

The specific detection steps are as follows:

*Step 1*: Fix the self-calibration theodolite on the lifting platform, install it on the air floating vibration isolation platform and level it. Set the horizontal angle of the self-calibration theodolite and the luminous theodolite to zero and the vertical angle to 90 degrees. Lock the horizontal and vertical adjustment knobs.

*Step 2*: Through the eyepiece of the self-calibrating theodolite, observe the cross wire emitted by the luminous theodolite, and the servo controller drives the two guide rails to slide until the cross wire of the luminous theodolite is aligned with the cross line of the self-calibrating theodolite.

*Step 3*: Taking the self-calibration theodolite as the reference, the horizontal angle and vertical angle of the luminous theodolite are given and rotated to this point to drive the displacement of the two guide rails. At the same time, the horizontal angle and vertical angle of the self-calibration theodolite are adjusted. The cross wire of the luminous theodolite is observed through the eyepiece of the self-calibration theodolite, and the adjustment is continued until the cross wire is aligned with the cross line of the self-calibration theodolite Reading and comparison, verify the alignment accuracy of the platform.

In the experiment, fifteen groups of horizontal angle *θ_y_*_1_ and vertical angle *θ_x_*_1_ of the luminous theodolite are given, and the horizontal angle *θ_y_*_2_ and vertical angle *θ_x_*_2_ of the self-calibration theodolite are recorded at the same time. According to Equation (9), the alignment errors Δ*θ_y_* and Δ*θ_x_* of the horizontal angle and vertical angle of the calibration platform are calculated respectively:

(9) $$\left\{\begin{array}{c} ∆\theta_{y}=\theta_{y1}-\theta_{y2}\\ ∆\theta_{x}=\frac{\theta_{x1}+\theta_{x2}}{2}-90{}^{\circ} \end{array}\right.$$

**Figure 14:**
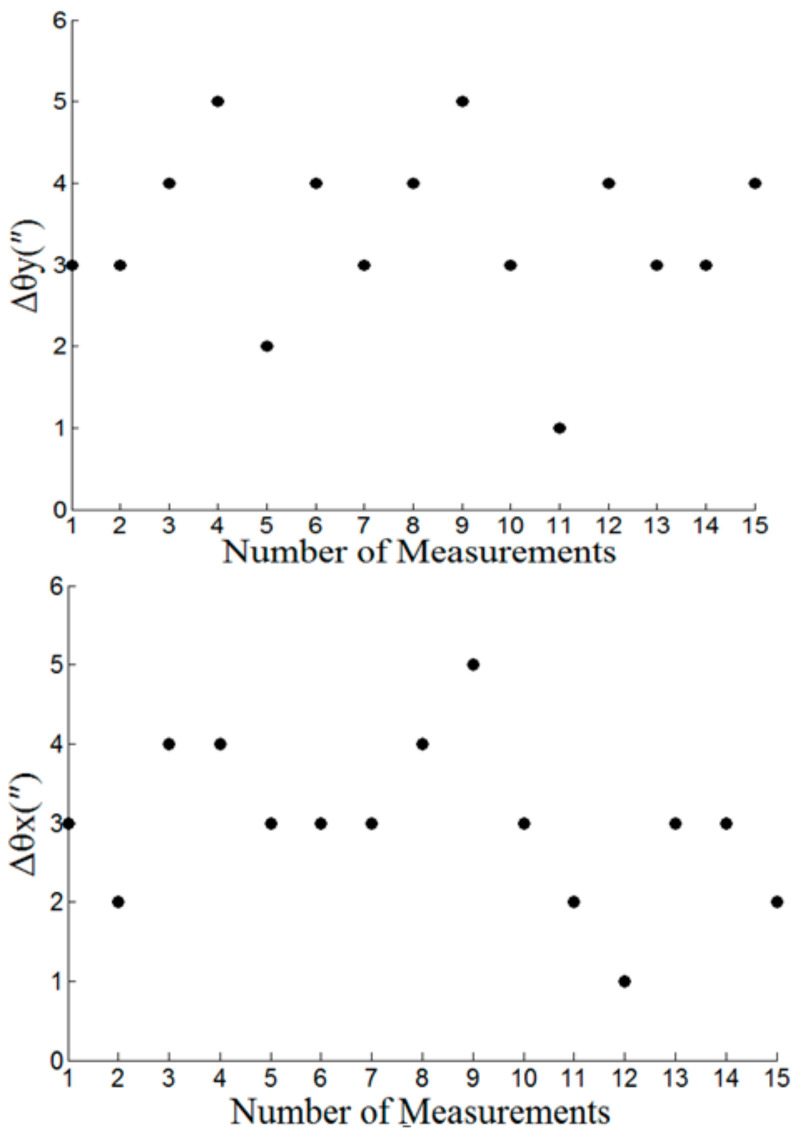
Alignment error testing results for four-axes calibration table.

The alignment error results of horizontal angle and vertical angle of calibration platform are shown in Figure 14.

Experimental results show that installation error of two-axes translation within the angle compensation range of theodolite, four-axes calibration table has an alignment accuracy of 5 arc sec for both horizontal and vertical angle, which is able to calibrate any detector of multidetector mosaic imaging systems accurately.

### 5.3. Experiments for Stereo Matching and Depth Information Estimation

**Figure 15:**
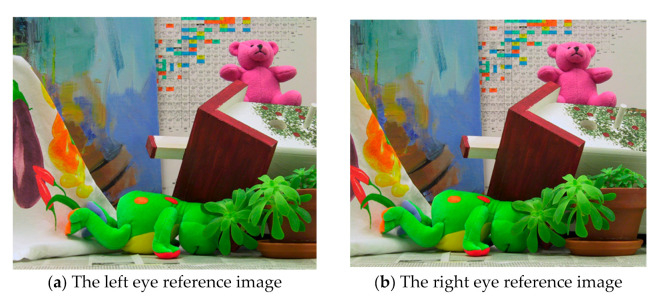
The left and right eye reference image of binocular original image.

**Figure 16:**
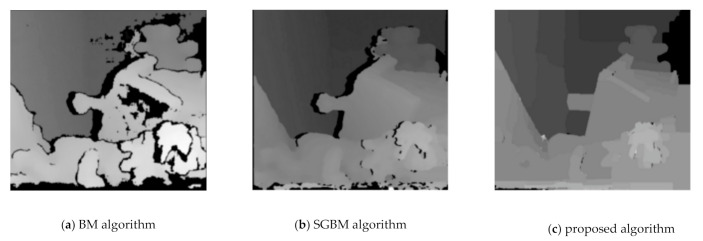
The disparity estimation results of BM, SGBM and the proposed algorithm.

After the camera is calibrated to get the internal and external parameters, in order to reduce the system error and error matching, we need to find the optimal stereo matching algorithm to improve the efficiency and accuracy of stereo matching. In order to verify the effectiveness of the algorithm, the proposed algorithm is compared with BM algorithms and SGBM algorithms [26]. The left and right eye reference image of binocular original image is shown in Figure 15. The disparity estimation results of the proposed algorithm, BM algorithm and SGBM algorithm are shown in Figure 16. The experimental results show that the parallax calculation effect of the proposed algorithm is smoother than that of BM algorithm and SGBM algorithm, and the noise is significantly reduced.

In addition, the comparison results of stereo matching efficiency and accuracy between the proposed algorithm, BM algorithm and SGBM algorithm are shown in Table 1, twenty groups of images are randomly selected from the adjacent cameras of the panoramic stereo imaging system to form a test sample data set, the running time, mean absolute error (MAE), root mean square error (RMSE) and mismatch percentage of the binocular stereo matching algorithm are calculated. It can be seen from Table 1 that the algorithm proposed in this paper runs faster, and the MAE and RMSE are also acceptable. As a global matching algorithm, the stereo matching effect of SGBM is obviously better than that of local matching algorithm, but at the same time, the complexity of SGBM is far greater than that of local matching algorithm.

**Figure 17:**
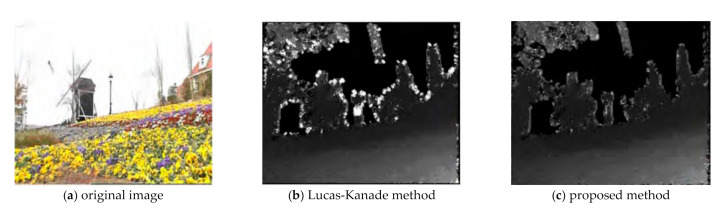
Depth images extracted by different optical flow methods.

By taking several groups of binocular images with known actual distance, a target is selected, and the distance between the target and the camera is actually measured. Then stereo matching is performed on the left and right reference images, and the pyramid Lucas-Kanade optical flow method based on image segmentation is used to extract the depth information. In depth estimation, the original pyramid Lucas-Kanade optical flow algorithm is compared with the pyramid Lucas-Kanade optical flow algorithm based on image segmentation. It can be seen from Figure 17 that the quality of the depth map obtained by the proposed algorithm is significantly improved, which is shown as follows: (1) using the image segmentation information to remove the bad points in the motion vector, so that the bad points in the depth map are significantly reduced. (2) The most frequent pixels of the same class are assigned to all pixels of the same class, which makes the scene edge information clearer and the obtained depth image less blocky.

We use pyramid Lucas-Kanade optical flow method based on image segmentation to extract depth information. The results and error analysis are shown in Table 2. Table 2 shows the estimated depth values of left and right cameras respectively. It can be seen from the comparison results in Table 2 that the positioning accuracy of binocular vision is high, and there is still a certain error between the estimation result and the actual depth. The reason may be that there is an error in the camera calibration data, or the matching accuracy is not very accurate. In practical application, the matching accuracy can be improved through preprocessing to further improve the accuracy of depth information estimation.

The adaptive iteration number method mentioned in this paper makes the iteration number of each layer of the optical flow method to estimate the depth information slightly different and reduces the time cost under the condition that the quality of the obtained depth image is almost the same. The average time of each frame of video can be saved by 39.4%.

### 5.4. Experiments for Panorama Mosaic

**Figure 18:**
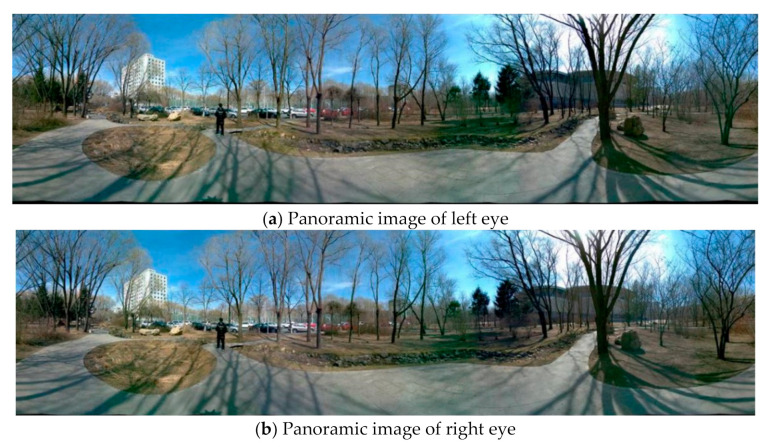
The left (**a**) and right eye (**b**) compose a stereoscopic panorama in good light.

**Figure 19:**
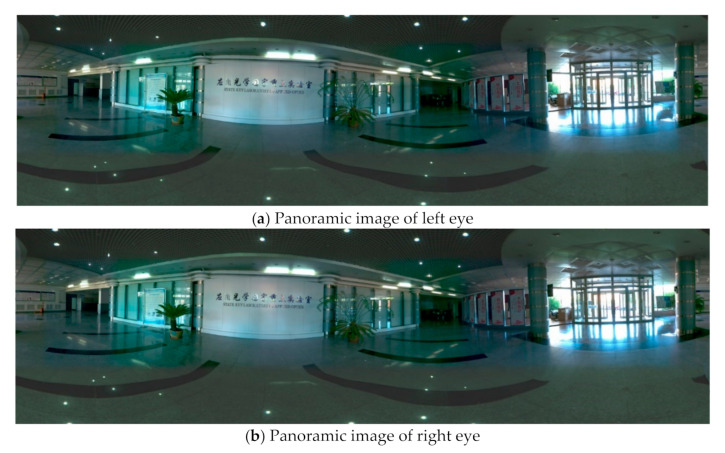
The left (**a**) and right eye (**b**) compose a stereoscopic panorama in uneven illumination.

**Figure 20:**
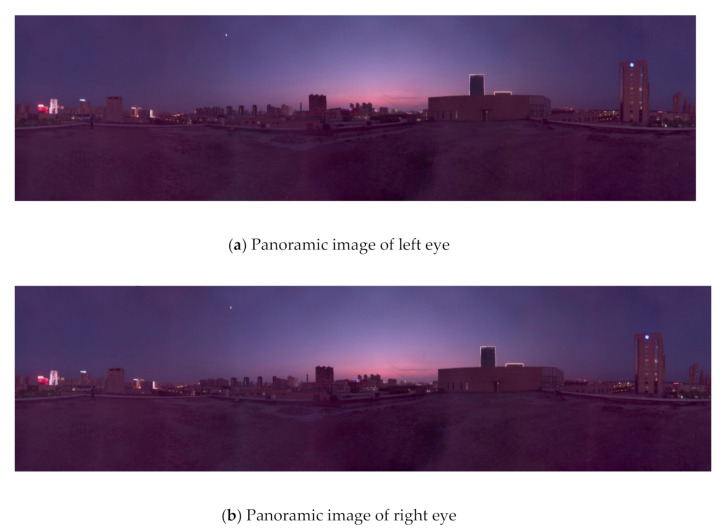
The left (**a**) and right eye (**b**) compose a stereoscopic panorama in weak light.

Panoramic mosaic imaging includes image preprocessing, feature detection and extraction, feature matching and pre-screening, and parameter estimation of registration transformation. At present, most players support up-down and left-right placement of stereo images. Because we are panoramic stereo, we place the left and right eye panoramic images in the stereo panoramic images in the up-down manner. The stereo images in different directions are stitched to get the stereo panorama of left and right eyes, as shown in Figure 18, Figure 19 and Figure 20.

From Figure 18 to Figure 20, it can be seen the imaging system can meet the requirements of clear imaging under different light conditions, also shows good performance for star level imaging under ultra-low illumination environment. According to the national optical and mechanical quality supervision and inspection center, the effective pixel of the imaging system is 30 million, the frame rate is 30 FPS, and the minimum illumination is 0.0051 lux.

Finally, 20 images are randomly selected to synthesize panoramic stereo images. The proposed algorithm and contrast algorithm [27] are used to calculate the image registration time, translation error and rotation error respectively. The average value of the calculation results is taken. The comparison of image registration change method and data is shown in Table 3.

Experimental results indicate that the algorithm has some invariance about the size, rotation and illumination changes, and the feature detector and matching time is 0.542 s, the registration transform time is 0.031 s, the registration error precision is less than 0.1 pixel, which can meet the requirements of the imaging system about the image registration including good real-time and accuracy performance, and has a valuable to engineering application.

## 6. Conclusions

In this paper, we mainly study the geometric model based on binocular stereo vision, and discuss the imaging principle of stereo vision. Firstly, the optical optimization design scheme of panoramic imaging based on binocular stereo vision is proposed, and the space coordinate calibration platform of an ultra-high precision panoramic camera based on theodolite angle compensation function is constructed. The projection matrix of adjacent cameras is obtained by solving the imaging principle of binocular stereo vision. Then, a real-time registration algorithm of multi-detector mosaic image and the Lucas-Kanade optical flow method based on image segmentation are proposed to realize stereo matching and depth information estimation of panoramic imaging, and the estimation results are analyzed effectively. Experimental results show that the proposed binocular panoramic stereo imaging system can meet the requirements of real-time and accuracy of image processing in virtual reality engineering applications.

## Figures and Tables

**Table 1:** Comparison of matching algorithms.

Matching Method	Running Time (ms)	MAE	RMSE	Mismatch Percentage (%)
**BM**	32	5.5432	12.9204	4.61
**SGBM**	76	5.5041	12.9892	3.82
**proposed**	21	3.1827	10.6416	2.93

**Table 2:** Comparison of depth estimation at different distances.

The Actual Distance between the Target Object and the Camera (mm)	The Depth Information Estimated by the Algorithm Is Proposed (mm)	Average Error between Calculated Value and Actual Value (%)
**1200**	1268.43 1263.31	5.21
	1258.24 1260.34	
**1800**	1842.95 1832.17	1.49
	1821.52 1810.99	
**2400**	2428.70 2420.01	0.52
	2410.01 2391.62	
**3000**	3012.52 3012.52	2.79
	3297.91 3012.52	

**Table 3:** Comparison of panoramic mosaic algorithms.

Algorithm	Registration Time (s)	Translation Error (Pixel)	Rotation Error (°)
**Contrast**	0.164	0.032	0.051
**proposed**	0.031	0.017	0.026

## Data Availability

The study did not report any data.
